# The effects of time-restricted eating on sleep in adults: a systematic review of randomized controlled trials

**DOI:** 10.3389/fnut.2024.1419811

**Published:** 2024-07-29

**Authors:** Carly Bohlman, Christian McLaren, Armin Ezzati, Patricia Vial, Daniel Ibrahim, Stephen D. Anton

**Affiliations:** ^1^Department of Physiology and Aging, College of Medicine, University of Florida, Gainesville, FL, United States; ^2^Department of Clinical and Health Psychology, University of Florida, Gainesville, FL, United States; ^3^Department of Food, Nutrition, Dietetics, and Health, Kansas State University, Manhattan, KS, United States

**Keywords:** time-restricted eating, sleep, intermittent fasting, time-restricted feeding, circadian rhythm, randomized control trials, meal timing, systematic review

## Abstract

**Introduction:**

Time-restricted eating (TRE), a dietary pattern reducing the duration of daily food consumption, has recently gained popularity. Existing studies show the potential benefits of TRE for cardiometabolic health. Uncertainty remains about whether these benefits are solely from altered meal timing or influences on other health behaviors, including sleep. Despite growing scientific interest in the effects of TRE on sleep parameters, the topic has not been systematically explored.

**Methods:**

This review examined the effects of TRE interventions (daily fasting duration ≥14 h) lasting at least 8 weeks on objective and subjective sleep parameters. Six randomized control trials were identified through Pubmed, Embase, Google Scholar, and Scopus through September 2023.

**Results:**

Of the included studies, three employed objective sleep measures using wearables and five studies assessed sleep subjectively through self-report questionnaires. Only one study reported significant improvements in subjective sleep quality following a TRE intervention. Additionally, one study found significant decreases in sleep duration, two studies found significant decreases in sleep efficiency, and one found significant increases in sleep onset latency.

**Discussion:**

Current evidence indicates that short to mid-term TRE does not typically worsen sleep parameters. However, some populations may experience reduced sleep disturbances, while others may experience reductions in sleep efficiency. Longer duration studies with objective sleep assessments are needed to better understand the effects of TRE on sleep parameters.

## 1 Introduction

The circadian system is responsible for various physiological processes over a 24-h cycle, including the sleep/wake cycle, heart rate, blood pressure, and hormone secretion (1). Accumulating evidence indicates that the disruption of the circadian rhythm is the cause of many metabolic diseases and is linked with sleep disturbances (2, 3). Despite the critical role sleep has in maintaining overall health (4–7), a growing percentage of adults report challenges falling and/or staying asleep. Specifically, more than 14% of adults indicate difficulty falling asleep, while 17% experience challenges in staying asleep (8). As of the year 2022, the CDC has reported that 36.8% of adults are reporting insufficient sleep which includes getting less than the recommended 7 h of sleep per night (9). This is of concern as long-term sleep disturbances increase the risk of chronic disease conditions, including diabetes, cardiovascular disease, hypertension, and obesity (10–12). Subsequently, improving sleep quality through increased sleep duration, regulated sleep schedules, and lowered sleep disruptions have been proposed to promote optimal health and quality of life, and reduce overall risk factors related to chronic conditions (13–15).

In line with this, sleep and meal timing have both been directly linked with both central and peripheral circadian rhythm, suggesting a common factor underlying the relationship between sleep quality and the timing of food intake (16). The energy metabolism pathway is circadian, affecting cellular function and metabolism of carbohydrates, proteins, and fat. As such, the use and storage of these macronutrients is not a linear process. In humans, insulin sensitivity varies according to the time of day, with decreased values in the evening and at night (17). In a study by Van Cauter et al., participants were observed in a sleep laboratory following a meal at the start of the study and then exposed to both uninterrupted sleep and interrupted sleep on different nights (17). During sleep deprivation, both glucose levels and insulin secretion rose to approximately the same level that corresponded to the beginning of the habitual sleep period. Additionally, daytime sleep was associated with a rise of 16% in glucose levels, 55% in insulin secretion, and 39% in serum insulin. This study demonstrates the importance that sleep quality and sleep timing both have on insulin and glucose regulation. Another study conducted by Arasaradnam et al. examined lipoprotein lipase activity in healthy adults before and after eating (18). This study revealed that peripheral circadian rhythms played a role in the activation of hormone-sensitive lipase (HSL), which contributes to nocturnal lipid intolerance. Specifically, participants with nightly fasting durations of 11.20 or longer displayed almost double the amplitude in HSL activity rhythm than those with short duration. Moreover, participants who habitually had early diners had 1.6 times higher amplitude than those who reported typically having late diners (18).

The relationship between meal timing and sleep has been examined in a few other human studies. For example, a cross-sectional study involving college-aged individuals found an association between the time in which the last meal was consumed with sleep efficiency. Specifically, individuals who consumed their last meal within 3 h of their bedtime had more frequent nighttime awakenings, and thus decreased sleep efficiency (19). In line with this, another study observed that food intake 30–60 min before sleep was associated with negative effects on sleep quality, as measured by polysomnography, in healthy men and women (16). Furthermore, another cross-sectional study, which looked at Japanese young adults showed that shorter sleep to dinner time windows were associated with an increase in sleep onset latency (20). Such findings suggest that altering the time in which we eat the last meal of the day can help align the body’s natural peripheral circadian rhythms, which could have a beneficial effect on the sleep–wake cycle and improve various health outcomes (21, 22). Findings from preclinical studies have also highlighted the importance of aligning the timing of food intake with the body’s natural circadian rhythm to produce improvements in sleep outcomes (23, 24).

A novel approach to altering the time in which food is eaten is called time-restricted eating (TRE), a type of intermittent fasting that typically involves restricting daily eating periods to 8–10 h and fasting for the remaining hours of the day and night. TRE has been proposed to promote healthy sleep patterns by aligning food intake with the body’s sleep/wake cycle (25). Potential mechanisms that may underlie the purported health benefits of TRE include its influence on peripheral circadian rhythms and autophagy processes (26). Emerging evidence suggests that metabolic switching, specifically the transition from utilizing glucose to ketone bodies as primary fuel substrates, may play a role in regulating circadian rhythms and contribute to the favorable cardiometabolic outcomes associated with TRE interventions (27).

Although this dietary approach has shown positive effects on body weight and overall metabolic health (25), its effect on sleep parameters is less understood. Animal studies have shown that time-restricted feeding (TRF) has a positive effect on sleep and health, by aligning food intake with the animal’s circadian rhythm, which is needed to maintain a robust sleep–wake cycle (28). In preclinical animal models, TRF, without reducing caloric intake, has been shown to decrease the severity of metabolic diseases, and has also improved different metabolic parameters which indirectly support better sleep. Wang et al. (29) found that, in mice, TRF can prevent and reverse diet-induced dysfunction of the paraventricular thalamic nucleus and excessive sleepiness. These results are promising, as high-fat diet-induced obesity is a growing public health concern and excessive daytime sleepiness is a common symptom of this type of obesity.

Additionally, by limiting food intake to a specific time window, TRF helps maintain a robust circadian rhythm, which includes the sleep–wake cycle. In addition, TRF has been shown to improve different metabolic parameters which indirectly support better sleep (30). King et al. (31) demonstrated that by synchronizing circadian rhythms, TRF exerts protective effects in mice models of Alzheimer’s disease, indirectly resulting in better sleep patterns. Further preclinical research in disease progression assessed the effectiveness of a circadian intervention in a neurodegenerative disease, specifically Huntington’s, where TRF implementation produced promising results in sleep improvements in male mice (32).

Current evidence from non-randomized human trials has produced mixed results. Specifically, a 14:10 TRE intervention (daily fasting for 14 and 10 h of eating) resulted in improved morning restfulness but had no effect on sleep quality in patients with metabolic syndrome after 12 weeks (33). Conversely, another trial involving 14:10 TRE improved sleep quality in overweight individuals after 16 weeks (34). Moreover, a shorter-term 8-week trial involving 9-h TRE had no effect on self-reported sleep duration in adults with obesity (35).

Such mixed findings suggest that TRE interventions produce varying results on sleep, with potential improvements for sleep parameters in some populations and deficits for others. A previous systematic review has explored the effects of another type of intermittent fasting, specifically Ramadan fasting, a type of religious, dry diurnal fasting in which individuals abstain from consuming foods and water from sundown to sunset, on sleep (36). The key finding of this review was that sleep duration was decreased during the Ramadan fasting month. However, this type of fasting is distinct from traditional TRE interventions in that food and liquids are only consumed during the evening and pre-dawn hours, rather than during the daytime.

The effects that TRE interventions have on sleep, particularly based on findings of randomized clinical trials (RCTs), however, have not been systematically examined. Thus, there is a need to systematically explore the effect that TRE has on sleep parameters based on the findings of RCTs. Therefore, this systematic review aimed to comprehensively summarize the current state of evidence of TRE interventions on measures of sleep based on findings of RCTs.

## 2 Materials and methods

The present systematic review used PRISMA (Preferred Reporting Items for Systematic Reviews and Meta-Analyses) guidelines for reporting methods and results (37).

### 2.1 Eligibility criteria

The present review contains RCTs that examined the effects of TRE on sleep parameters. Studies with the following characteristics were included: (1) Study design: randomized controlled trials in adults (≥18 y) with a minimum duration of 8 weeks, (2) Measured sleep parameters (sleep duration, sleep efficiency, sleep onset latency) and/or subjective sleep quality, (3) Language: English, (4) Publication year: studies published from September 2008 until September 2023 (the last 15 years) as research on time-restricted eating has only recently gained more attention, (5) TRE protocol (daily fasting ≥14 h). Studies were excluded if they prescribed <14 h fasting, included types of intermittent fasting other that TRE (e.g., Ramadan fasting, alternative day fasting, or fasting mimicking diet), did not measure sleep quality, had a duration <8 weeks, or were published in a language other than English.

### 2.2 Search strategy

The authors (CB, CM) conducted two separate searches on PubMed, Embase, Google Scholar, and Scopus from the date of March 2023 until September 2023. The search terms that we used included *time-restricted eating* or *time-restricted feeding* or *intermittent fasting* and *sleep* or *Pittsburgh sleep quality index* or *sleep duration* or *sleep efficiency* or *sleep onset latency*. For each database, keywords and MeSH terms were searched within the title/abstract, and subject/keywords. Additionally, filters were applied as they were available on each database. Filters included: (1) English, (2) clinical trial, (3) randomized controlled trial, (4) humans, (5) 2008–2023. Content experts (SA, AE) were incorporated into the review process to provide expertise and rigor. The detailed search strategy used for each database is shown in Supplementary material.

### 2.3 Study selection

Independent reviewers (CB, CM) assessed each record for the prior inclusion or exclusion criterion based on an initial review of the title and abstract. Articles that met all the inclusion criteria were retrieved for review of the full text of the article, those that showed any of the exclusion criteria were not considered. Citations retrieved from the initial database search were imported into Zotero version 6.0.30 for the purposes of duplicate screening, and full-text article review. Each citation underwent blinded screening by two independent reviewers, with any discrepancies resolved through reviewer consensus. The same independent reviewers assessed the full-text articles separately before a joint assessment of the articles. Discrepancies regarding the inclusion of an article were resolved through subsequent discussions, resulting in a mutual decision by the reviewers.

### 2.4 Data extraction

Extraction of relevant data was completed by two independent reviewers (CB, DI). Data that was extracted was related to the characteristics of the included study, specifically including study design, recruitment setting, edibility criteria, and population characteristics. These reviewers extracted the data from the included studies, the data was assessed and any disputes that arose were resolved through discussions between reviewers to determine mutual agreement.

### 2.5 Subjective and objective sleep measurements

In addition to examining study characteristics, this review also included both objective and subjective sleep measurements. Objective sleep measurements refer to data collected independently of human reporting. Studies included in this review utilize objective measurements, obtained using accelerometer-based wearables, such as the Oura ring and the ActiGraph watch. These non-invasive devices monitor rest and activity patterns by recording movements in three dimensions and tracking frequency, intensity, and duration. Algorithms process this data to determine sleep and wake periods, where minimal movement indicates sleep and increased movement indicates activity. These measurements provide sleep duration, sleep efficiency, and sleep onset latency. In addition to objective data, subjective sleep quality was assessed through self-report questionnaires. The Pittsburgh Sleep Quality (PSQI) was used to gather participants’ retrospective evaluations of their sleep. These questionnaires assess various aspects of sleep, including perceived sleep quality, bedtime, and wake time.

Subjective measures, while less precise, offer valuable insights into the perception of sleep quality and disturbances. The included studies included the use of the PSQI, a retrospective self-report questionnaire. The PSQI has been extensively validated and shown to be reliable in clinical settings (38). Studies have demonstrated its correlation with objective sleep measures, distinguishing between “good” and “poor” sleepers, and confirming good internal consistency and test–retest reliability (39–41).

For objective measures of sleep, the included studies employed actigraphy devices. Actigraphy utilizes wearable technology, making it particularly suitable for clinical trials aiming to objectively measure sleep. Actigraphy has been validated against polysomnography (PSG), the gold standard in objective sleep measurement, and is recognized for its validity in community settings (42). It provides reliable data across different settings, enhancing our understanding of sleep outcomes in diverse populations (43, 44).

### 2.6 Baseline sleep quality status

The baseline sleep quality status of participants enrolled in each study was determined through assessment of baseline PSQI scores by independent reviewers. Participants who scored <5 were classified as having good sleep quality, while those who scored >5 were categorized as poor sleepers. A PSQI score of 5 represents the cutoff range for clinically significant sleep disturbances and this method aligns with established practices in sleep research (45).

### 2.7 Quality assessment

For quality assessment of the included studies, the Cochrane Risk of Bias Tool for Randomized Trials was utilized (46). Two independent reviewers (CB, CM) determined the risk of bias (RoB) of each of the included articles. The judgment of articles consisted of categorizing, as low risk of bias, some concern, or high risk of bias, over five domains of bias, including the following: randomization process, deviation from the intended intervention, outcome data, measurement of the outcome, and selection of the reported results.

## 3 Results

### 3.1 Search results

**Figure 1:**
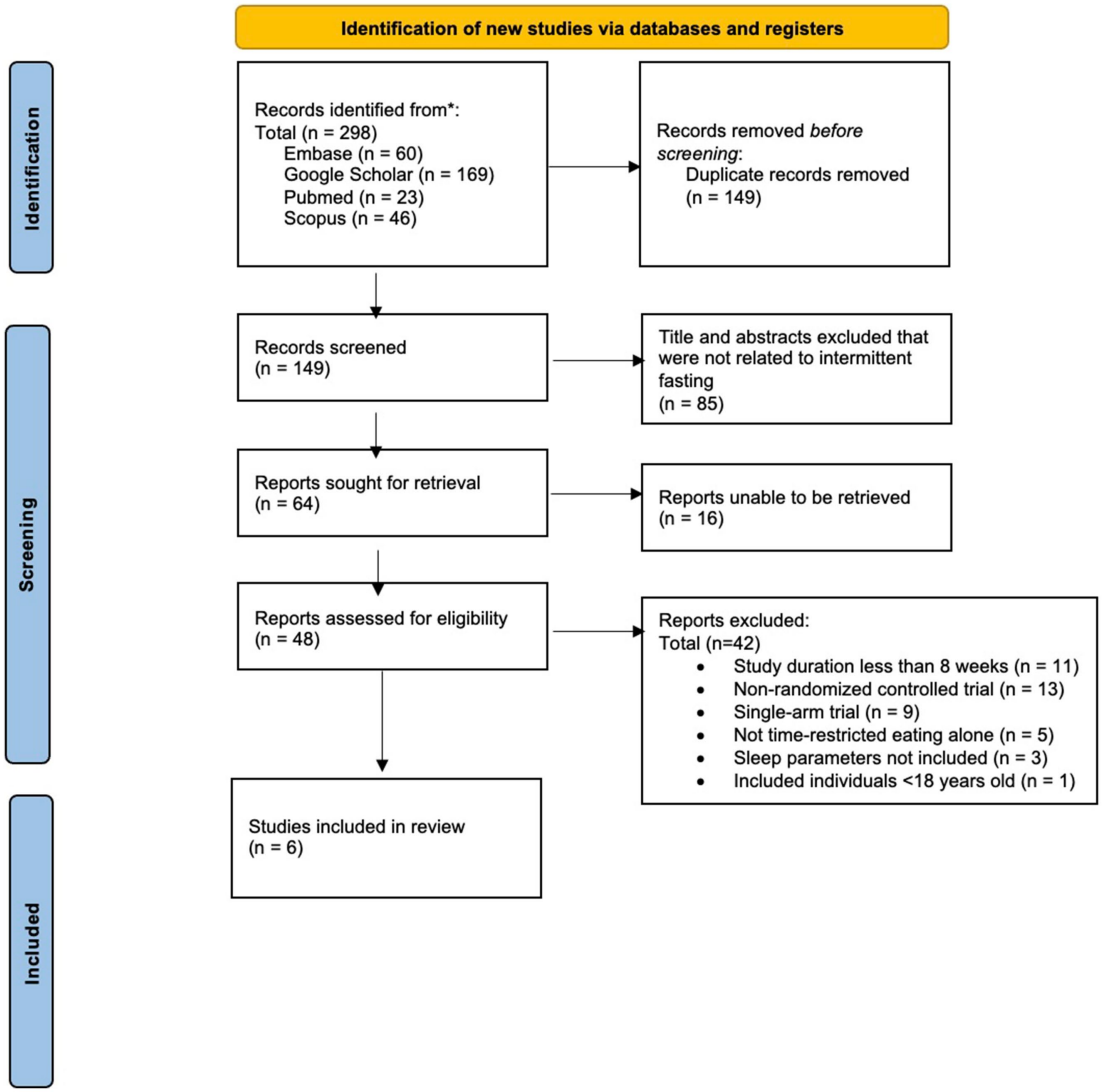
PRISMA flow chart.

A comprehensive literature search identified a total of 298 abstracts for initial review (see Figure 1).

After removing duplicate publications (*n* = 149), 149 unique abstracts remained. The analysis of abstracts and titles led to the exclusion of a total of 85 articles, leaving 64 reports for full-text retrieval. Among these, 16 articles were inaccessible due to articles not being digitalized and licensing issues, rendering them ineligible for inclusion. Subsequently, a thorough examination of the 48 retrieved full-text articles was conducted, resulting in the exclusion of 42 articles due to non-randomized study designs, and/or outcome measures not including sleep parameters. Thus, out of the initial pool of 149 articles screened, six articles met our predetermined eligibility criteria and were included in the present systematic review.

### 3.2 Study characteristics

Four of the included studies recruited adults with overweight or obesity (47–50). The two remaining studies encompassed specific populations, including young adults (51) and 24-h shift workers (52). There was a total of 548 participants who were initially enrolled across these studies, with 430 completing the research interventions, representing a 21.5% attrition rate across all studies included.

The mean age of participants across all included studies ranged from 22 to 49 years, representing both young and middle-aged populations. The intervention durations ranged from 8 to 14 weeks, and the daily fasting duration within TRE interventions varied from 14 to 20 h. The daily fasting window was determined either based on participant choice or dictated explicitly by the study protocol. Among the six included studies, one study employed a 14:10 TRE intervention (52), three studies employed a 16:8 TRE intervention (47, 49, 50), one study employed an 18:6 TRE intervention (51), and one study implemented a 20:4 TRE intervention (48). Only one study implemented a TRE protocol with the addition of calorie restriction (CR), a strategy aimed at reducing calories in order to reduce body weight (50). The utilization of a sleep quality assessment as a criterion for participant eligibility was not applied across any of the included studies. Table 1 provides a summary of key characteristics from the six eligible studies included in this systematic review.

**Table 1:** Characteristics of included studies.

Study	Country	Sample size	Key eligibility criteria	Age (y), mean ± SD	Intervention	Eating window	Duration	Sleep measurement
Lowe et al. (47)	USA	141	Adults with overweight and obesity; good and poor sleepers	Total: 46.5 ± 10.5 dTRE: 46.8 ± 10.8 CMT: 46.1 ± 10.3	dTRE	12:00 p.m. – 8:00 p.m.	12 weeks	Objective: Oura ring Subjective: PSQI
					CMT	3 structured meals throughout the day		
Manoogian et al. (52)	USA	150	Adult 24-h shift workers with cardiometabolic risk factors; poor sleepers	Total: 40.4 ± 9.4 TRE: 41.1 ± 8.7 SOC: 40.0 ± 9.4	TRE	Self-selected eating period between 9:00 am - 7:00 pm; 10-h fasting period	12 weeks	Objective: Actiwatch Subjective: PSQI, ESS
					SOC	*Ad libitum*		
Cienfuegos et al. (48)	USA	58	Adults with obesity; poor sleepers	4-h dTRE: 49.0 ± 2.0 6-h dTRE: 46.0 ± 3.0 Control: 45.0 ± 2.0	4-h dTRE	3:00 p.m. – 7:00 p.m.	8 weeks	Objective: N/A Subjective: PSQI
					6-h dTRE	1:00 p.m. – 7:00 p.m.		
					Control	*Ad libitum*		
Simon et al. (49)	USA	20	Adults with obesity; unreported good and/or poor sleepers	TRE: 46.4 ± 12.4 Non-TRE: 44.2 ± 12.3	TRE	Self-selected eating window; 8 h	12 weeks	Objective: ActiGraph Link Subjective: N/A
					Non-TRE	Typical eating habits		
Steger et al. (50)	USA	90	Adults with obesity; good and poor sleepers	Total: 44 ± 12 eTRE: 46 ± 11 Control: 42 ± 12	eTRE	7:00 a.m. – 3:00 p.m.	14 weeks	Objective: N/A Subjective: PSQI
					Control	Self-selected eating window; 12-h window		
Zhang et al. (51)	China	89	Young adults with overweight and obesity; unreported good and/or poor sleepers	eTRE: 23.8 ± 0.6 dTRE: 23.2 ± 0.5 Control: 22.1 ± 0.4	eTRE	7:00 a.m. – 1:00 p.m.	10 weeks	Objective: N/A Subjective: PSQI
					dTRE	12:00 p.m. to 6:00 p.m.		
					Control	*Ad libitum*		

TRE, time-restricted eating; CMT, consistent meal timing; dTRE, delayed time-restricted eating; eTRE, early time-restricted eating; TRF, time-restricted feeding; SOC, standard of care; PSQI, Pittsburgh Sleep Quality Index; ESS, Epworth Sleepiness Scale.

### 3.3 Risk of bias

**Figure 2:**
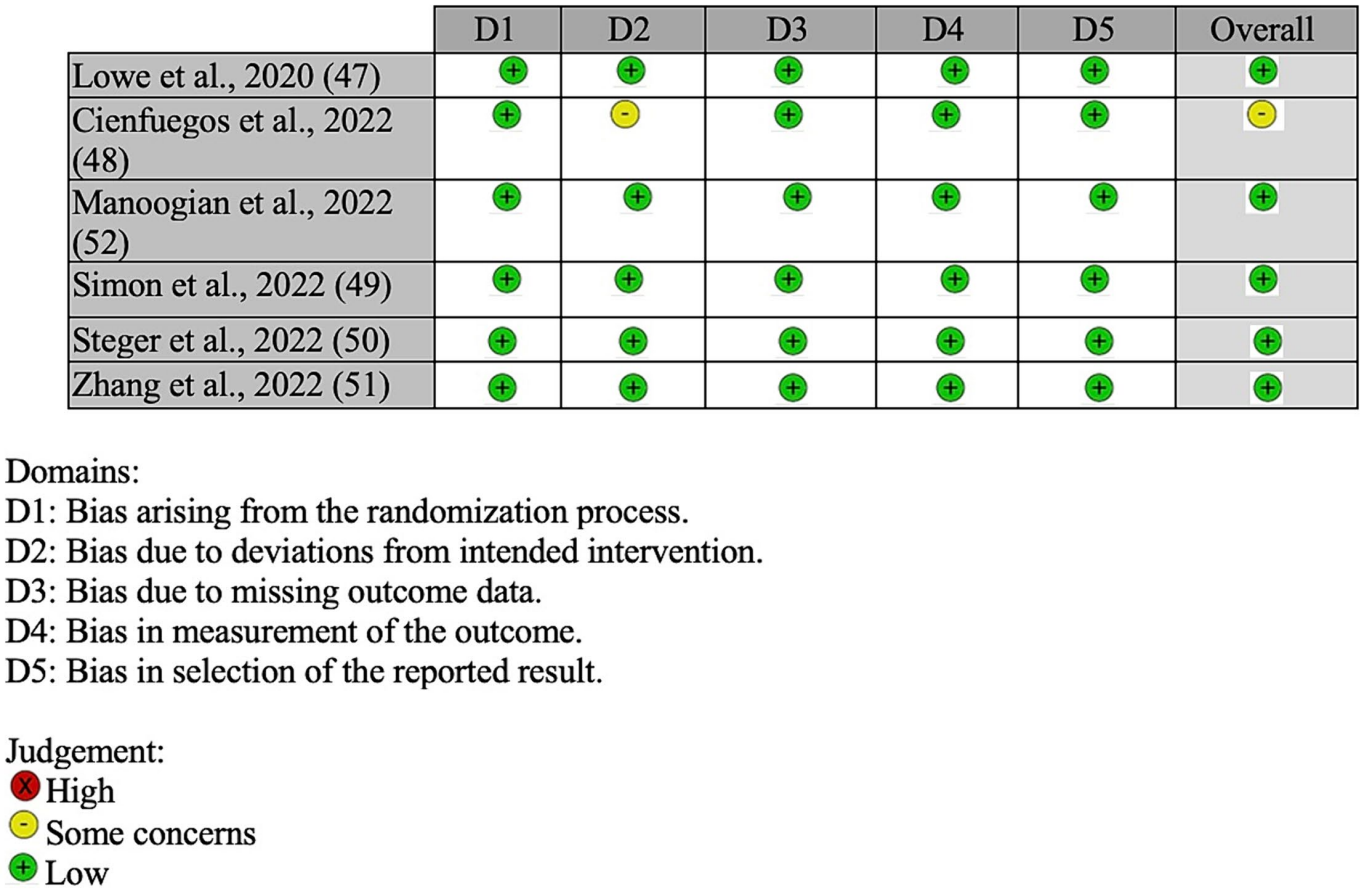
The risk of bias assessment summary for the included studies.

Of the six included studies, five studies were determined to be of a low risk of having bias (47, 49–52) and one as having some risk of bias (48) (see Figure 2). For bias arising from the randomization process, all six studies were determined to have a low risk. For bias due to deviations of intended procedures, five studies were determined to have a low risk (47, 49–52), while one study had some risk due to potential bias to deviations from intended interventions (48). For bias resulting from missing data, measurement of outcome, and selection of reported results, all six studies were determined to have a low risk.

### 3.4 Sleep measurements

Among the six studies that were included in this systematic review, three studies employed wearable sleep measurement devices for measuring sleep duration, efficiency, and onset latency (47, 49, 52), while the remaining three studies (48, 50, 51) relied on participant self-report. Measurements were gathered at baseline and following the completion of the intervention employed in each study.

#### 3.4.1 Sleep quality

Five of the six included studies utilized subjective sleep quality (47–51) using the PSQI, with one study (52) additionally incorporating the use of the Epworth Sleep Scale (ESS). Only one (52) of the five studies that measured sleep quality found a statistically significant outcome, showing a significant decrease in the number of self-reported sleep disturbances observed within the TRE group (14:10 TRE) from the baseline to the post-intervention assessments. None of the other four studies (47, 48, 50, 51) reported statistically significant changes in PSQI or ESS scores from baseline following participation in a TRE intervention.

#### 3.4.2 Sleep duration

Five of the six included studies assessed sleep duration (48–52), with two utilizing objective measurement methods (49, 52) and two relying on self-reported data (48, 50, 51). No statistically significant results were observed in studies using objective measurement methods. However, one study utilizing self-reported sleep quality yielded a significant result. Steger et al. (50) reported a significant decrease in mean self-reported sleep duration by 0.5 h from baseline (*p* < 0.05) over the 14-week early TRE (eTRE) intervention. This reduction in sleep duration within the eTRE group was significantly different from the control group, which reported an increase in sleep duration of 17 min (*p* < 0.05).

#### 3.4.3 Sleep efficiency

Three of the included studies assessed sleep efficiency (47, 50, 52), with two utilizing objective sleep measurement methods (47, 52) and one relying on participant self-report (50). Of these, two studies (47, 50), one with objective measures and one with subjective measures, reported significant reductions in sleep efficiency in the TRE intervention groups. Lowe et al. (47) reported a significant decrease in mean sleep efficiency of 2.7%, measured by the Oura ring, for the TRE group (late TRE; eating window between 12:00 p.m. – 8:00 p.m.) from baseline (*p* < 0.05), which was also significantly different from the control group (*p* < 0.05). Steger et al. (50) found a significant decrease in mean self-reported PSQI sleep efficiency of 2% (SD 1%) for the eTRE group (eating window between 7:00 a.m. – 3:00 p.m.) from baseline (*p* < 0.05), which was also significantly different from the control (*p* < 0.05).

#### 3.4.4 Sleep onset latency

Four of the included studies (47, 48, 50, 52) measured sleep onset latency, with one utilizing objective sleep measurement methods and three relying on participant self-report. Of these, only one study showed a significant change based on participant report (50). Steger et al. (50) reported a significant increase in self-reported sleep onset latency of 7 min within the eTRE group from baseline (*p* < 0.05), which was statistically different from the control group (*p* < 0.05).

## 4 Discussion

The purpose of this systematic review was to examine the current state of evidence on the effects of TRE on sleep parameters in adults. Based on objective measures, TRE interventions had minimal effects on sleep parameters and typically did not exhibit a worsening effect in the included studies. However, one 12-week study observed a significant reduction in sleep efficiency based on objective measurements obtained by the Oura ring with late TRE compared to a control condition in adults with overweight or obesity (47). When subjective measures were used, the effects were mixed. In one 14-week study testing the effects of eTRE in adults with obesity, participants reported reductions in sleep duration and efficiency, and a small increase in sleep onset latency (50). In contrast, participants who were 24-h shift workers reported reductions in self-reported sleep disturbances following a 12-week self-selected TRE (14:10) intervention (52). Thus, our findings suggest that short-term TRE interventions may improve some aspects of sleep quality in individuals with circadian disruption (24-h shift workers) but may have a negative effect on sleep duration and efficiency in other populations.

The variations between studies in terms of fasting protocols, participant baseline sleep status, and study durations may explain the mixed effects. Of note, two studies (49, 52) had self-selected eating windows, two studies (50, 51) specified early or late TRE, and two studies had pre-selected eating windows that were not considered early or late TRE (47, 48). Notably, of the six included studies, two studies (47, 50) included both good and poor sleepers, and two studies (48, 52) exclusively enrolled poor sleepers as determined through PSQI scores at baseline. The remaining two studies did not report baseline PSQI values (51) or did not utilize the PSQI assessment (49).

Based on objective measures, sleep efficiency was decreased in one study (47), during which participants were asked to limit their eating windows to later in the day (12:00 p.m. – 8:00 p.m.) for 12 weeks. This study utilized the Oura ring while the other two studies (49, 52) in which sleep parameters remained unchanged employed the Actiwatch (52) or ActiGraph tools (49). The reason for the mixed results is unclear, however, the type of measurement device used may have influenced the results due to different sleep/wake measures within wearable technology. For self-reported sleep efficiency, only one study (50) that included both good and poor sleepers in the eTRE intervention group found statistically significant decreases in sleep efficiency. Importantly, these effects were not significant enough to reclassify individuals with initially good sleep as poor sleepers following the intervention. Therefore, the effects on sleep efficiency were not clinically significant.

A significant reduction in self-reported sleep duration was reported in only one study compared to the control arm (50). Steger et al. (50) implemented an 8-h eating window with an eTRE intervention combined with CR with a duration of 14 weeks. The addition of CR to the TRE intervention, as well as the longer study duration, may have resulted in significant changes in sleep parameters in this study (50). In contrast, shorter trials that imposed TRE with *ad libitum* intake did not alter sleep durations in studies with both poor and good sleepers (47, 49, 51). Similarly, sleep onset latency was only found to be significantly increased in the study by Steger et al. (50), and it is possible that the addition of CR to the TRE intervention may have contributed to this significant outcome.

In line with our findings, circadian rhythm regulation has emerged as a pivotal mechanism through which TRE is thought to regulate the sleep/wake cycle, affecting peripheral clocks and synchronizing metabolic processes, thereby optimizing sleep architecture (26). It is hypothesized that metabolic switching, which is the transition from utilizing glucose to ketone bodies as primary fuel substrates, may play a role in regulating circadian rhythm (53) This metabolic transition triggers various cellular responses including reduced levels of leptin and insulin, the mobilization of fatty acids, and increased adiponectin levels (54) Circadian alignment through the reinforcement of daily fasting/eating windows by TRE may consequently enhance sleep quality (26).

Notably, in a randomized controlled trial assessing gene expression analysis, TRE altered the expression of six genes associated with circadian rhythm (55). Likewise, skipping dinner in TRE protocols (eTRE) significantly reduced cortisol levels in the evening suggesting the impact of TRE on the circadian rhythm of cortisol levels (55). By contrast, findings from previous systematic reviews suggest that Ramadan fasting may disturb circadian rhythm by significantly decreasing sleep duration (36), and melatonin secretion during Ramadan month (56). Similarly, shift work-induced circadian rhythm disruption induces sleep disturbances most likely due to irregular sleeping schedules or patterns (57).

The bi-directional relationship between circadian misalignment and sleep disruption and/or irregularity may be altered through the timing of food intake (58, 59). This further supports the hypothesis that alignment of food intake in TRE protocols is the key determinant of its impact on hormonal regulation and sleep outcomes. In line with this, a study included in our review by Manoogian et al. observed reductions in self-reported nightly sleep disturbances, which suggested improved sleep quality following a 14:10 TRE intervention (52). Noteworthy, this study included a population of 24-h shift workers (mostly men; 91%) who experience limited or unpredictable sleep schedules and thus they may be more likely than other populations to benefit from a TRE pattern. The findings of this study are specific to a population with circadian misalignment, however, and thus may not apply to all populations. Noteworthy, sleep quality was not affected in the other five studies included in this review.

Findings from this systematic review should be interpreted with caution. It must be noted that study-specific factors, such as participant levels of physical activity or diet and/or self-report, may have influenced the included study data. An important limitation of this review is the scarce number of studies on individuals with circadian disturbances which limited our findings. Our findings were unable to encompass the strength of the effects of TRE on sleep in populations with circadian misalignment due to the limited number of published studies. Many studies on TRE are conducted in populations with overweight or obesity, narrowing the findings of our review to specific populations. Furthermore, inability to retrieve 16 articles that were identified as relevant to our research. The primary reasons included the lack of digital versions and access restrictions due to licensing issues. This limitation may have affected the comprehensiveness of our literature review and potentially influenced our findings. Another limitation of this review is that fasting initiating times across the included studies were not comparable, which could make it challenging to draw conclusions on the effects of TRE on circadian rhythm and sleep. Additionally, not all included studies assessed the same sleep measures, and the methods used to assess these varied across studies. Specifically, only three (47, 49, 52) of the identified studies employed objective measures of sleep duration, efficiency, and onset latency. In contrast, the remaining studies (48, 50, 51) depended on self-report methodologies, utilizing sleep questionnaires, mainly the PSQI. The use of self-reported sleep measurements may introduce a potential for participant response bias, a factor that warrants consideration in the interpretation of subjective findings. Lastly, the studies included in this review had a relatively short duration, ranging from 8 to 14 weeks, thus the long-term effects of TRE on sleep parameters cannot be concluded from this review.

This systematic review also had a few noteworthy strengths. To our knowledge, this was the first time that the effects of TRE on sleep have been systematically explored. Furthermore, all included studies were RCTs with a duration of at least 8 weeks and daily fasting periods of a minimum of 14 h. Across all included studies, there was a 78% completion rate with minimal reported adverse outcomes. Additionally, the studies were of high quality with relatively low to moderate risk of bias.

Future studies should assess the effects of TRE on sleep disturbances due to circadian misalignment to further explore the proposed relationship between circadian alignment, TRE, and sleep. More specifically, studies should consider the effects of meal timing on objective and subjective sleep parameters in individuals with good or poor sleep quality as separate populations. In comparing preclinical and clinical studies, there are vast differences in fasting protocol which should be considered when creating future studies. Preclinical models tend to implement shorter fasting durations to assess immediate fasting effects on biological markers that could suggest results in human models (60). Future research should determine the optimal fasting duration and initiation times for improving sleep outcomes. Moreover, given that accumulating evidence suggests aging is associated with disruption of circadian rhythms (61), future studies should investigate the effects of TRE on sleep disturbances in older adults. Findings from observational studies indicate the association between longer fasting hours in TRE regimen (≥12 h fasting) with significant improvement in sleep duration in older adults (62); however, there is a lack of evidence from clinical trials investigating the potential role that TRE can play in improving sleep in the older population. Additionally, studies should include longer TRE interventions to examine the long-term effects of meal timing on sleep quality. Finally, it is recommended that future studies incorporate self-reported sleep measures alongside wearable sleep-tracking devices, as this dual approach will allow for a comprehensive assessment of alterations in sleep parameters following a TRE intervention.

## 5 Conclusion

The findings from the present systematic review suggest that short to mid-term TRE interventions in adults generally do not influence objective or subjective sleep quality. TRE interventions, however, may improve some aspects of perceived sleep quality in individuals with circadian rhythm disruptions. In other populations, however, short to mid-term TRE interventions have the potential to reduce sleep duration and efficiency, as well as increase sleep onset latency. Thus, further research with longer study durations is needed to fully understand the complex interactions between meal timing, circadian rhythms, and their specific impacts on sleep outcomes.

## Data availability statement

The original contributions presented in the study are included in the article/Supplementary material, further inquiries can be directed to the corresponding author.

## Author contributions

CB: Conceptualization, Data curation, Formal analysis, Investigation, Methodology, Visualization, Writing – original draft, Writing – review & editing. CM: Conceptualization, Data curation, Formal analysis, Funding acquisition, Investigation, Methodology, Visualization, Writing – original draft, Writing – review & editing. AE: Formal analysis, Investigation, Methodology, Writing – original draft, Writing – review & editing. PV: Formal analysis, Investigation, Methodology, Writing – original draft, Writing – review & editing. DI: Data curation, Writing – original draft, Writing – review & editing. SA: Conceptualization, Writing – original draft, Writing – review & editing.

## References

[1] Longo VD Panda S. Fasting, circadian rhythms, and time-restricted feeding in healthy lifespan. Cell Metab. (2016) 23:1048–59. doi: 10.1016/j.cmet.2016.06.001, PMID: 27304506 PMC5388543

[2] Adafer R Messaadi W Meddahi M Patey A Haderbache A Bayen S . Food timing, circadian rhythm and Chrononutrition: a systematic review of time-restricted Eating's effects on human health. Nutrients. (2020) 12:3770. doi: 10.3390/nu12123770, PMID: 33302500 PMC7763532

[3] Khan S Malik BH Gupta D Rutkofsky I. The role of circadian misalignment due to insomnia, lack of sleep, and shift work in increasing the risk of cardiac diseases: a systematic review. Cureus. (2020) 12:e6616. doi: 10.7759/cureus.6616, PMID: 32064196 PMC7008727

[4] Watson NF Badr MS Belenky G Bliwise DL Buxton OM Buysse D . Joint consensus statement of the American Academy of sleep medicine and Sleep Research Society on the recommended amount of sleep for a healthy adult: methodology and discussion. J Clin Sleep Med. (2015) 11:931–52. doi: 10.5664/jcsm.4950, PMID: 26235159 PMC4513271

[5] Triantafillou S Saeb S Lattie EG Mohr DC Kording KP. Relationship between sleep quality and mood: ecological momentary assessment study. JMIR Mental Health. (2019) 6:e12613. doi: 10.2196/12613, PMID: 30916663 PMC6456824

[6] Bhatt P Patel V Motwani J Choubey U Mahmood R Gupta V . Insomnia and cardiovascular health: exploring the link between sleep disorders and cardiac arrhythmias. Curr Cardiol Rep. (2023) 25:1211–21. doi: 10.1007/s11886-023-01939-x, PMID: 37656386

[7] Deak MC Stickgold R. Sleep and cognition. Wiley Interdiscip Rev Cogn Sci. (2010) 1:491–500. doi: 10.1002/wcs.52, PMID: 26271496 PMC5831725

[8] Adjaye-Gbewonyo D Ng AE Black LI. Sleep difficulties in adults: United States, 2020. NCHS Data Brief. (2022) 436:1–8. doi: 10.15620/cdc:117490 35792564

[9] Centers for Disease Control and Prevention (2024). FastStats: sleep in adults. Available at: https://www.cdc.gov/sleep/data-research/facts-stats/adults-sleep-facts-and-stats.html?CDC_AAref_Val=https://www.cdc.gov/sleep/data-and-statistics/adults.html

[10] Ramar K Malhotra RK Carden KA Martin JL Abbasi-Feinberg F Aurora RN . Sleep is essential to health: an American Academy of sleep medicine position statement. J Clin Sleep Med. (2021) 17:2115–9. doi: 10.5664/jcsm.9476, PMID: 34170250 PMC8494094

[11] Grandner MA . Sleep, health, and society. Sleep Med Clin. (2022) 17:117–39. doi: 10.1016/j.jsmc.2022.03.001 35659068

[12] Consensus Conference Panel Watson NF Badr MS Belenky G Bliwise DL Buxton OM . Joint consensus statement of the American Academy of sleep medicine and Sleep Research Society on the recommended amount of sleep for a healthy adult: methodology and discussion. Sleep. (2015) 38:1161–83. doi: 10.5665/sleep.4886, PMID: 26194576 PMC4507722

[13] Henst RHP Pienaar PR Roden LC Rae DE. The effects of sleep extension on cardiometabolic risk factors: a systematic review. J Sleep Res. (2019) 28:e12865. doi: 10.1111/jsr.12865, PMID: 31166059

[14] Leggett A Assari S Burgard S Zivin K. The effect of sleep disturbance on the association between chronic medical conditions and depressive symptoms over time. Longit Life Course Stud. (2017) 8:138–51. doi: 10.14301/llcs.v8i2.433, PMID: 28966664 PMC5617341

[15] Lauderdale DS Chen JH Kurina LM Waite LJ Thisted RA. Sleep duration and health among older adults: associations vary by how sleep is measured. J Epidemiol Community Health. (2016) 70:361–6. doi: 10.1136/jech-2015-206109, PMID: 26530811 PMC4788566

[16] Crispim CA Zimberg IZ dos Reis BG Diniz RM Tufik S de Mello MT. Relationship between food intake and sleep pattern in healthy individuals. J Clin Sleep Med. (2011) 7:659–64. doi: 10.5664/jcsm.1476, PMID: 22171206 PMC3227713

[17] Van Cauter E Blackman JD Roland D Spire JP Refetoff S Polonsky KS. Modulation of glucose regulation and insulin secretion by circadian rhythmicity and sleep. J Clin Invest. (1991) 88:934–42. doi: 10.1172/JCI115396, PMID: 1885778 PMC295490

[18] Arasaradnam MP Morgan L Wright J Gama R. Diurnal variation in lipoprotein lipase activity. Ann Clin Biochem. (2002) 39:136–9. doi: 10.1258/0004563021901883 11928761

[19] Chung N Bin YS Cistulli PA Chow CM. Does the proximity of meals to bedtime influence the sleep of young adults? A cross-sectional survey of university students. Int J Environ Res Public Health. (2020) 17:2677. doi: 10.3390/ijerph17082677, PMID: 32295235 PMC7215804

[20] Yasuda J Kishi N Fujita S. Association between time from dinner to bedtime and sleep quality indices in the young Japanese population: a cross-sectional study. Die Dermatol. (2023) 2:140–9. doi: 10.3390/dietetics2020011

[21] Hepler C Weidemann BJ Waldeck NJ Marcheva B Cedernaes J Thorne AK . Time-restricted feeding mitigates obesity through adipocyte thermogenesis. Science. (2022) 378:276–84. doi: 10.1126/science.abl8007, PMID: 36264811 PMC10150371

[22] Almeneessier AS Alzoghaibi M BaHammam AA Ibrahim MG Olaish AH Nashwan SZ . The effects of diurnal intermittent fasting on the wake-promoting neurotransmitter orexin-a. Ann Thorac Med. (2018) 13:48–54. doi: 10.4103/atm.atm_181_17, PMID: 29387256 PMC5772108

[23] Huang W Ramsey KM Marcheva B Bass J. Circadian rhythms, sleep, and metabolism. J Clin Invest. (2011) 121:2133–41. doi: 10.1172/JCI46043, PMID: 21633182 PMC3104765

[24] Potter GD Skene DJ Arendt J Cade JE Grant PJ Hardie LJ. Circadian rhythm and sleep disruption: causes, metabolic consequences, and countermeasures. Endocr Rev. (2016) 37:584–608. doi: 10.1210/er.2016-1083 27763782 PMC5142605

[25] Parr EB Devlin BL Hawley JA. Perspective: time-restricted eating-integrating the what with the when. Adv Nutr Bethesda. (2022) 13:699–711. doi: 10.1093/advances/nmac015, PMID: 35170718 PMC9156382

[26] Ezzati A Pak VM. The effects of time-restricted eating on sleep, cognitive decline, and Alzheimer's disease. Exp Gerontol. (2023) 171:112033. doi: 10.1016/j.exger.2022.112033, PMID: 36403899

[27] Anton SD Moehl K Donahoo WT Marosi K Lee SA Mainous AG 3rd . Flipping the metabolic switch: understanding and applying the health benefits of fasting. Obesity. (2018) 26:254–68. doi: 10.1002/oby.22065, PMID: 29086496 PMC5783752

[28] Manoogian ENC Chow LS Taub PR Laferrère B Panda S. Time-restricted eating for the prevention and Management of Metabolic Diseases. Endocr Rev. (2022) 43:405–36. doi: 10.1210/endrev/bnab027, PMID: 34550357 PMC8905332

[29] Wang X Xing K He M He T Xiang X Chen T . Time-restricted feeding is an intervention against excessive dark-phase sleepiness induced by obesogenic diet. Natl Sci Rev. (2022) 10:nwac222. doi: 10.1093/nsr/nwac222 36825118 PMC9942665

[30] Deota S Panda S. New horizons: circadian control of metabolism offers novel insight into the cause and treatment of metabolic diseases. J Clin Endocrinol Metab. (2021) 106:e1488–93. doi: 10.1210/clinem/dgaa691, PMID: 32984881 PMC7947830

[31] King MW Chen Y Musiek ES. Time-restricted feeding and Alzheimer's disease: you are when you eat. Trends Mol Med. (2023) 29:974–5. doi: 10.1016/j.molmed.2023.10.004, PMID: 37872024 PMC10842495

[32] Chiem E Zhao K Dell'Angelica D Ghiani CA Paul KN Colwell CS. Scheduled feeding improves sleep in a mouse model of Huntington's disease. bioRxiv. (2024). doi: 10.1101/2024.05.04.592428, PMID: 38284849 PMC11973937

[33] Wilkinson MJ Manoogian ENC Zadourian A Lo H Fakhouri S Shoghi A . Ten-hour time-restricted eating reduces weight, blood pressure, and Atherogenic lipids in patients with metabolic syndrome. Cell Metab. (2020) 31:92–104.e5. doi: 10.1016/j.cmet.2019.11.004, PMID: 31813824 PMC6953486

[34] Hutchison AT Liu B Wood RE Vincent AD Thompson CH O'Callaghan NJ . Effects of intermittent versus continuous energy intakes on insulin sensitivity and metabolic risk in women with overweight. Obesity. (2019) 27:50–8. doi: 10.1002/oby.22345 30569640

[35] Gill S Panda S. A smartphone app reveals erratic diurnal eating patterns in humans that can be modulated for health benefits. Cell Metab. (2015) 22:789–98. doi: 10.1016/j.cmet.2015.09.005, PMID: 26411343 PMC4635036

[36] Faris MAE Jahrami HA Alhayki FA Alkhawaja NA Ali AM Aljeeb SH . Effect of diurnal fasting on sleep during Ramadan: a systematic review and meta-analysis. Sleep Breath. (2020) 24:771–82. doi: 10.1007/s11325-019-01986-1, PMID: 31832984

[37] Moher D Shamseer L Clarke M Ghersi D Liberati A Petticrew M . Preferred reporting items for systematic review and meta-analysis protocols (PRISMA-P) 2015 statement. Syst Rev. (2015) 4:1. doi: 10.1186/2046-4053-4-1, PMID: 25554246 PMC4320440

[38] Wang L Wu YX Lin YQ Wang L Zeng ZN Xie XL . Reliability and validity of the Pittsburgh sleep quality index among frontline COVID-19 health care workers using classical test theory and item response theory. J Clin Sleep Med. (2022) 18:541–51. doi: 10.5664/jcsm.9658, PMID: 34534069 PMC8805004

[39] Buysse DJ Reynolds CF 3rd Monk TH Berman SR Kupfer DJ. The Pittsburgh sleep quality index: a new instrument for psychiatric practice and research. Psychiatry Res. (1989) 28:193–213. doi: 10.1016/0165-1781(89)90047-4, PMID: 2748771

[40] Carpenter JS Andrykowski MA. Psychometric evaluation of the Pittsburgh sleep quality index. J Psychosom Res. (1998) 45:5–13. doi: 10.1016/s0022-3999(97)00298-5 9720850

[41] Mollayeva T Thurairajah P Burton K Mollayeva S Shapiro CM Colantonio A. The Pittsburgh sleep quality index as a screening tool for sleep dysfunction in clinical and non-clinical samples: a systematic review and meta-analysis. Sleep Med Rev. (2016) 25:52–73. doi: 10.1016/j.smrv.2015.01.009, PMID: 26163057

[42] Thomas J. S. Gamble K. (2024). Actigraphy in the evaluation of sleep disorders—UpToDate. Available at: https://www.uptodate.com/contents/actigraphy-in-the-evaluation-of-sleep-disorders

[43] Marino M Li Y Rueschman MN Winkelman JW Ellenbogen JM Solet JM . Measuring sleep: accuracy, sensitivity, and specificity of wrist actigraphy compared to polysomnography. Sleep. (2013) 36:1747–55. doi: 10.5665/sleep.3142, PMID: 24179309 PMC3792393

[44] Sadeh A . The role and validity of actigraphy in sleep medicine: an update. Sleep Med Rev. (2011) 15:259–67. doi: 10.1016/j.smrv.2010.10.001, PMID: 21237680

[45] Zhong QY Gelaye B Sánchez SE Williams MA. Psychometric properties of the Pittsburgh sleep quality index (PSQI) in a cohort of Peruvian pregnant women. J Clin Sleep Med. (2015) 11:869–77. doi: 10.5664/jcsm.4936, PMID: 25845902 PMC4513264

[46] Higgins JP Altman DG Gøtzsche PC Jüni P Moher D Oxman AD . The Cochrane Collaboration's tool for assessing risk of bias in randomised trials. BMJ. (2011) 343:d5928. doi: 10.1136/bmj.d5928 22008217 PMC3196245

[47] Lowe DA Wu N Rohdin-Bibby L Moore AH Kelly N Liu YE . Effects of time-restricted eating on weight loss and other metabolic parameters in women and men with overweight and obesity: the TREAT randomized clinical trial. JAMA Intern Med. (2020) 180:1491–9. doi: 10.1001/jamainternmed.2020.4153, PMID: 32986097 PMC7522780

[48] Cienfuegos S Gabel K Kalam F Ezpeleta M Pavlou V Lin S . The effect of 4-h versus 6-h time restricted feeding on sleep quality, duration, insomnia severity and obstructive sleep apnea in adults with obesity. Nutr Health. (2022) 28:5–11. doi: 10.1177/02601060211002347, PMID: 33759620 PMC8460695

[49] Simon SL Blankenship J Manoogian ENC Panda S Mashek DG Chow LS. The impact of a self-selected time restricted eating intervention on eating patterns, sleep, and late-night eating in individuals with obesity. Front Nutr. (2022) 9:1007824. doi: 10.3389/fnut.2022.1007824, PMID: 36337640 PMC9634110

[50] Steger FL Jamshed H Bryan DR Richman JS Warriner AH Hanick CJ . Early time-restricted eating affects weight, metabolic health, mood, and sleep in adherent completers: a secondary analysis. Obesity. (2023) 31 Suppl 1:96–107. doi: 10.1002/oby.23614, PMID: 36518092 PMC9877132

[51] Zhang LM Liu Z Wang JQ Li RQ Ren JY Gao X . Randomized controlled trial for time-restricted eating in overweight and obese young adults. iScience. (2022) 25:104870. doi: 10.1016/j.isci.2022.104870, PMID: 36034217 PMC9400087

[52] Manoogian ENC Zadourian A Lo HC Gutierrez NR Shoghi A Rosander A . Feasibility of time-restricted eating and impacts on cardiometabolic health in 24-h shift workers: the healthy heroes randomized control trial. Cell Metab. (2022) 34:1442–1456.e7. doi: 10.1016/j.cmet.2022.08.018 36198291 PMC9536325

[53] Peeke PM Greenway FL Billes SK Zhang D Fujioka K. Effect of time restricted eating on body weight and fasting glucose in participants with obesity: results of a randomized, controlled, virtual clinical trial. Nutr Diabetes. (2021) 11:6. doi: 10.1038/s41387-021-00149-0, PMID: 33446635 PMC7809455

[54] Mattson MP Longo VD Harvie M. Impact of intermittent fasting on health and disease processes. Ageing Res Rev. (2017) 39:46–58. doi: 10.1016/j.arr.2016.10.005, PMID: 27810402 PMC5411330

[55] Jamshed H Beyl RA Della Manna DL Yang ES Ravussin E Peterson CM. Early time-restricted feeding improves 24-hour glucose levels and affects markers of the circadian clock, aging, and autophagy in humans. Nutrients. (2019) 11:1234. doi: 10.3390/nu11061234, PMID: 31151228 PMC6627766

[56] Chawla S Beretoulis S Deere A Radenkovic D. The window matters: a systematic review of time restricted eating strategies in relation to cortisol and melatonin secretion. Nutrients. (2021) 13:2525. doi: 10.3390/nu13082525, PMID: 34444685 PMC8399962

[57] James SM Honn KA Gaddameedhi S Van Dongen HPA. Shift work: disrupted circadian rhythms and sleep-implications for health and well-being. Curr Sleep Med Rep. (2017) 3:104–12. doi: 10.1007/s40675-017-0071-6, PMID: 29057204 PMC5647832

[58] Liu J Yi P Liu F. The effect of early time-restricted eating vs later time-restricted eating on weight loss and metabolic health. J Clin Endocrinol Metab. (2023) 108:1824–34. doi: 10.1210/clinem/dgad036, PMID: 36702768

[59] Jamshed H Steger FL Bryan DR Richman JS Warriner AH Hanick CJ . Effectiveness of early time-restricted eating for weight loss, fat loss, and Cardiometabolic health in adults with obesity: a randomized clinical trial. JAMA Intern Med. (2022) 182:953–62. doi: 10.1001/jamainternmed.2022.3050 35939311 PMC9361187

[60] Rothschild J Hoddy KK Jambazian P Varady KA. Time-restricted feeding and risk of metabolic disease: a review of human and animal studies. Nutr Rev. (2014) 72:308–18. doi: 10.1111/nure.12104 24739093

[61] Buhr ED Takahashi JS. Molecular components of the mammalian circadian clock. Handb Exp Pharmacol. (2013) 217:3–27. doi: 10.1007/978-3-642-25950-0_1, PMID: 23604473 PMC3762864

[62] Estrada-deLeón DB Struijk EA Caballero FF Sotos Prieto M Rodríguez-Artalejo F Lopez-Garcia E. Prolonged nightly fasting and lower-extremity functioning in community-dwelling older adults. Br J Nutr. (2021) 126:1347–54. doi: 10.1017/S0007114520005218, PMID: 33371909 PMC8505711

