# Supplementary figures and images for: The effects of time-restricted eating on sleep in adults: a systematic review of randomized controlled trials
Source: Front Nutr. 2024 Jul 29;11:1419811. doi: 10.3389/fnut.2024.1419811 (PMC11322763; doi:10.3389/fnut.2024.1419811)

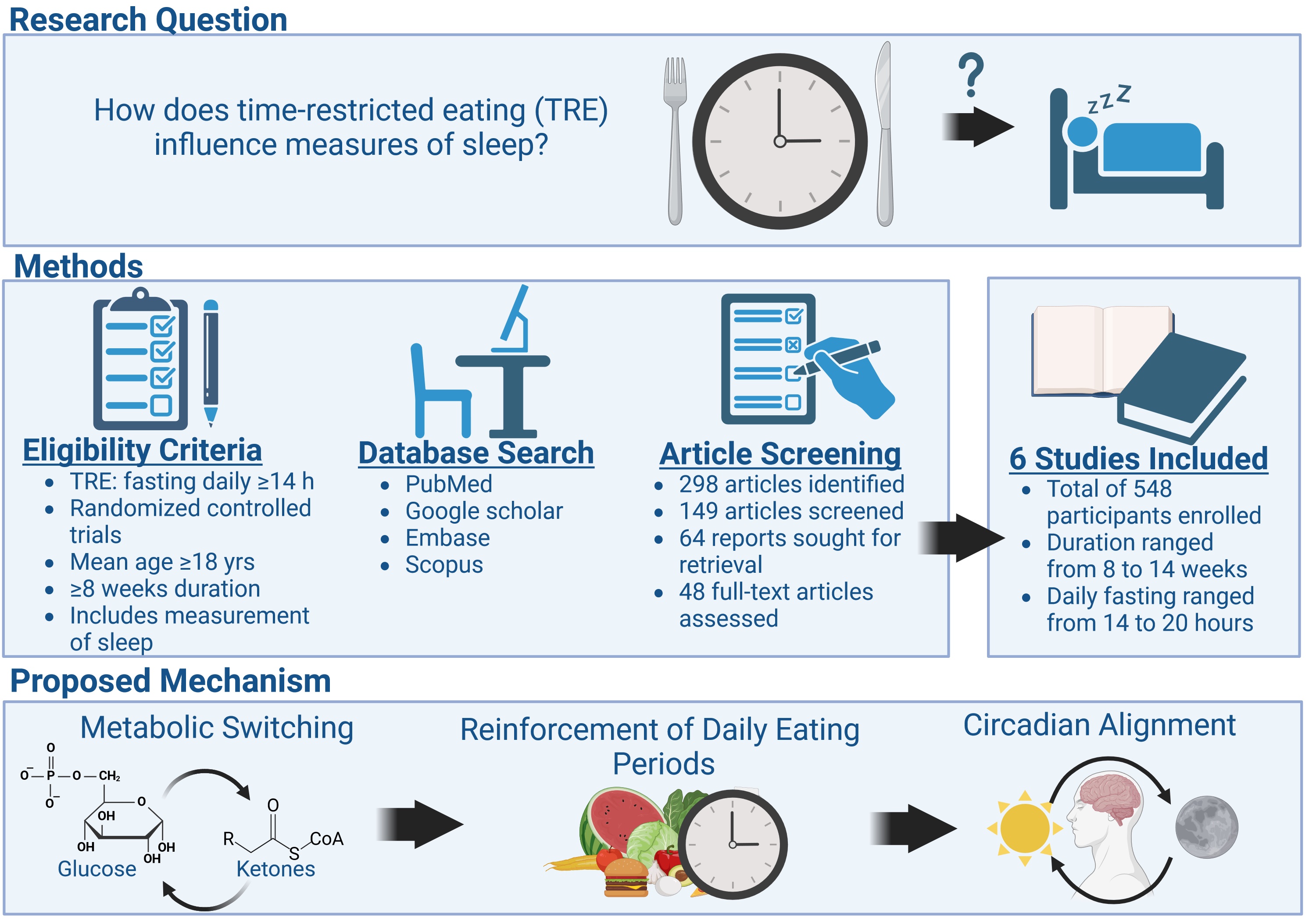

Supplement: Supplementary file 1 [file Image_1.JPEG]
